# Interferon-gamma release assay levels and risk of progression to active tuberculosis: a systematic review and dose-response meta-regression analysis

**DOI:** 10.1186/s12879-021-06141-4

**Published:** 2021-05-22

**Authors:** Jorge R. Ledesma, Jianing Ma, Peng Zheng, Jennifer M. Ross, Theo Vos, Hmwe H. Kyu

**Affiliations:** 1 Institute for Health Metrics and Evaluation, University of Washington, 3980 15th Ave. NE, Seattle, WA 98195 USA; 2 Department of Health Metrics Sciences, University of Washington, 3980 15th Ave. NE, Seattle, WA 98195 USA; 3 Department of Global Health, University of Washington, 325 9th Avenue, Box 359931, Seattle, WA 98104 USA; 4 Department of Medicine, University of Washington, 1959 NE Pacific Street, Box 356420, Seattle, WA 98195 USA

**Keywords:** Latent tuberculosis, IGRA, Active tuberculosis, Dose-response meta-regression

## Abstract

**Background:**

Identifying and treating individuals with high risk of progression from latent tuberculosis infection to active tuberculosis (TB) disease is critical for eliminating the disease. We aimed to conduct a systematic review and meta-regression analysis to quantify the dose-response relationship between interferon-gamma release assay (IGRA) levels and the risk of progression to active TB.

**Methods:**

We searched PubMed and Embase from 1 January 2001 to 10 May 2020 for longitudinal studies that reported the risk of progression from latent to active TB as a function of baseline IGRA values. We used a novel Bayesian meta-regression method to pool effect sizes from included studies and generate a continuous dose-response risk curve. Our modeling framework enabled us to incorporate random effects across studies, and include data with different IGRA ranges across studies. The quality of included studies were assessed using the Newcastle-Ottawa scale (NOS).

**Results:**

We included 34 studies representing 581,956 person-years of follow-up with a total of 788 incident cases of TB in the meta-regression analysis. Higher levels of interferon-gamma were associated with increased risk of progression to active tuberculosis. In the dose-response curve, the risk increased sharply between interferon-gamma levels 0 and 5 IU/ml, after which the risk continued to increase moderately but at a slower pace until reaching about 15 IU/ml where the risk levels off. Compared to 0 IU/ml, the relative risk of progression to active TB among those with interferon-gamma levels of 0.35, 1, 5, 10, 15, and 20 IU/ml were: 1.64 (1.28–2.08), 2.90 (2.02–3.88), 11.38 (6.64–16.38), 19.00 (13.08–26.90), 21.82 (14.65–32.57), and 22.31 (15.43–33.00), respectively. The dose-response relationship remains consistent when limiting the analysis to studies that scored highest in the NOS.

**Conclusion:**

The current practice of dichotomizing IGRA test results simplifies the TB infection disease continuum. Evaluating IGRA test results over a continuous scale could enable the identification of individuals at greatest risk of progression to active TB.

**Supplementary Information:**

The online version contains supplementary material available at 10.1186/s12879-021-06141-4.

## Background

Tuberculosis (TB) is the leading cause of mortality from a single infectious agent, with more than 1 million deaths per year [1]. *Mycobacterium tuberculosis* (Mtb) is the causative agent of TB, though a person with Mtb infection can remain asymptomatic in a state known as latent TB infection (LTBI). While individuals with LTBI are asymptomatic, they are an important reservoir for future TB cases. Approximately one quarter of the world’s population is estimated to have LTBI [2, 3], of which 5 to 15% will develop active TB at some point in their lives [4]. Identifying individuals with LTBI and placing those at risk of developing active TB on preventive treatment is thus critical for eliminating the disease [5].

The tuberculin skin test (TST) has traditionally been used to test for latent TB infection, but has known limitations. Most notably, its specificity is adversely affected by BCG vaccination or infection with nontuberculous mycobacteria. Additionally, TST requires multiple health care visits over 48–72 h to place the test and read the result, which may be prone to inter-reader variability [6]. Alternatives to the TST are T-cell-based interferon-gamma release assays (IGRAs). Two forms of IGRAs are currently available for commercial use that are widely used: QuantiFERON TB Gold in tube (QFT-GIT) and T-SPOT.TB. IGRAs have several advantages over TST such as higher specificity for Mtb and lack of cross reaction with BCG vaccination. IGRAs have become the primary diagnostic tool for LTBI in low to intermediate TB burden countries [7]. Despite the higher cost of the test and some challenges with reproducibility, the use of IGRAs is expected to further expand in high TB burden countries, as the World Health Organization (WHO) has recently endorsed the use of QFT-GIT and T-SPOT.TB for the End TB strategy [8].

Given the likely increased use of QFT-GIT in high TB burden settings, it is critical to review the utility of QFT-GIT to predict progression from latent to active TB. A recent meta-analysis of cohort studies indicated that TB contacts with a positive IGRA result have a 10.8-fold higher rate of progression to active TB [9]. In addition, recent individual studies have suggested a need to further examine the entire distribution of IGRA values for risk analyses of subsequent active TB [10–12]. These studies have shown a marked increase in the risk of incident active TB disease with higher IGRA values. Some researchers have called for a need to report a borderline zone, an intermediate area between a negative and positive IGRA test, to improve the diagnostic accuracy of potential development of active TB [13–15]. Together the literature suggests that there is potentially a loss of information for identification of individuals at high risk of active TB when IGRA results are dichotomized by the traditional 0.35 IU/ml threshold.

Previously published systematic reviews and meta-analyses of IGRA performance have yet to take into account the full spectrum of the infection by considering IGRA values at a continuous scale. A consideration of the entire distribution of IGRA levels rather than a binary cut-off may help inform treatment considerations for those at the highest risk of progressing to active TB. We aimed to conduct a systematic review and meta-analysis to quantify the dose-response relationship between IGRA levels and risk of progression to active TB using all available global data sources.

## Methods

### Literature search

In this systematic review and meta-regression analysis we followed the PRISMA and MOOSE checklists. We searched PubMed and Embase from 1 January 2001 to 10 May 2020 for studies that reported the risk of progression from latent to active TB based on baseline IGRA values. The full search strategy is available in the supplementary material. In addition, we made no restrictions in study language. The reference lists of eligible full texts and identified systematic reviews were reviewed for additional relevant studies.

### Study selection

Retrospective or prospective cohort studies and clinical trials that assessed QuantiFERON-TB Gold in tube (QFT-GIT) or QuantiFERON-TB Gold Plus (QFT-Plus) results, defined as the difference of interferon-gamma level between the TB antigen tube and negative control tube, as the exposure variable and progression to active tuberculosis as an outcome, were eligible for inclusion. The study participants were adults or children who were free of active TB disease at baseline. We excluded studies that contained a sample with a confounding disease (e.g. lung cancer, rheumatic diseases, inflammatory bowel diseases), that did not test all individuals with QuantiFERON-TB, or did not follow-up all individuals for progression to active TB disease. We further excluded studies that only focused on QuantiFERON-TB conversion or had participants with previous positive QuantiFERON-TB tests.

### Data extraction

Data were extracted using a standardized data extraction form developed a priori. The following variables were extracted from each study: study design, location, follow-up duration, sample attrition rate, baseline age-sex distribution, TB preventive treatment use, sample size by baseline QuantiFERON-TB results (IU/ml categories), number of cases progressing to active TB by IU/ml categories, and method for diagnosing active TB. Information on participant characteristics (e.g. general population, TB contacts, healthcare workers, etc.) was also extracted. In studies that separately reported data from participants who developed TB very early (under 2 months of the start of the study), they were considered as prevalent TB cases and were excluded during extraction such that they did not contribute to the sample size for baseline QuantiFERON-TB results nor for the number of incident TB cases. When available, extracted data were stratified by TB preventive treatment use. For person-year information, we used the study mean or median as follow-up time if follow-up time was not disaggregated for every QuantiFERON-TB category.

### Assessment of quality of included studies

We examined the quality of included studies using the Newcastle-Ottawa quality assessment scale (NOS) [16]. Potential scores range from 0 to 9 points, with higher scores representing higher quality studies. Study scores were based on the following criteria: selection of the study population, comparability between exposed and non-exposed groups, and assessment of the outcome.

### Data analysis

Incidence rate ratios (referred to as relative risks in this paper) for each study were computed by using the total number of cases that progressed to active TB and accumulated person-time information for each IGRA category. We then used a novel Bayesian meta-regression method [17] to analyze data from included studies and generate a continuous risk curve for the association between IGRA values and risk of progression to active TB. This method allowed us to incorporate random-effects across studies and include heterogeneous data with various IGRA categories. The primary advantage of this method is that it is able to take any IGRA range (e.g. 0–0.35, 0.35–1, 4–10, 0.35–20, etc.) as input and use integration techniques to derive continuous risks over the entire distribution of IGRA values. Detailed methods and equations can be found in the supplementary material. We separately analyzed studies where the study population were people living with HIV (PLHIV).

### Sensitivity analyses and subgroup analyses

We assessed the robustness of the dose-response risk curve by conducting a sensitivity analysis stratifying results by their NOS score. We assessed for the potential of effect modification by study-level population. The population subgroup analyses included: TB case contacts, healthcare workers, migrants, PLHIV, adults (> 18 years), and children (< 18 years). We conducted additional subgroup analyses by stratifying results by whether studies provided any TB preventive treatment (TPT) or not. Across the subgroup analyses, all model parameters for the Bayesian meta-regression remained consistent with the primary analysis. To investigate differences in the dose-response curves between subgroups we generated 1000 estimates from the posterior distributions to generate a ratio of the relative risks with uncertainty.

## Results

**Fig. 1:**
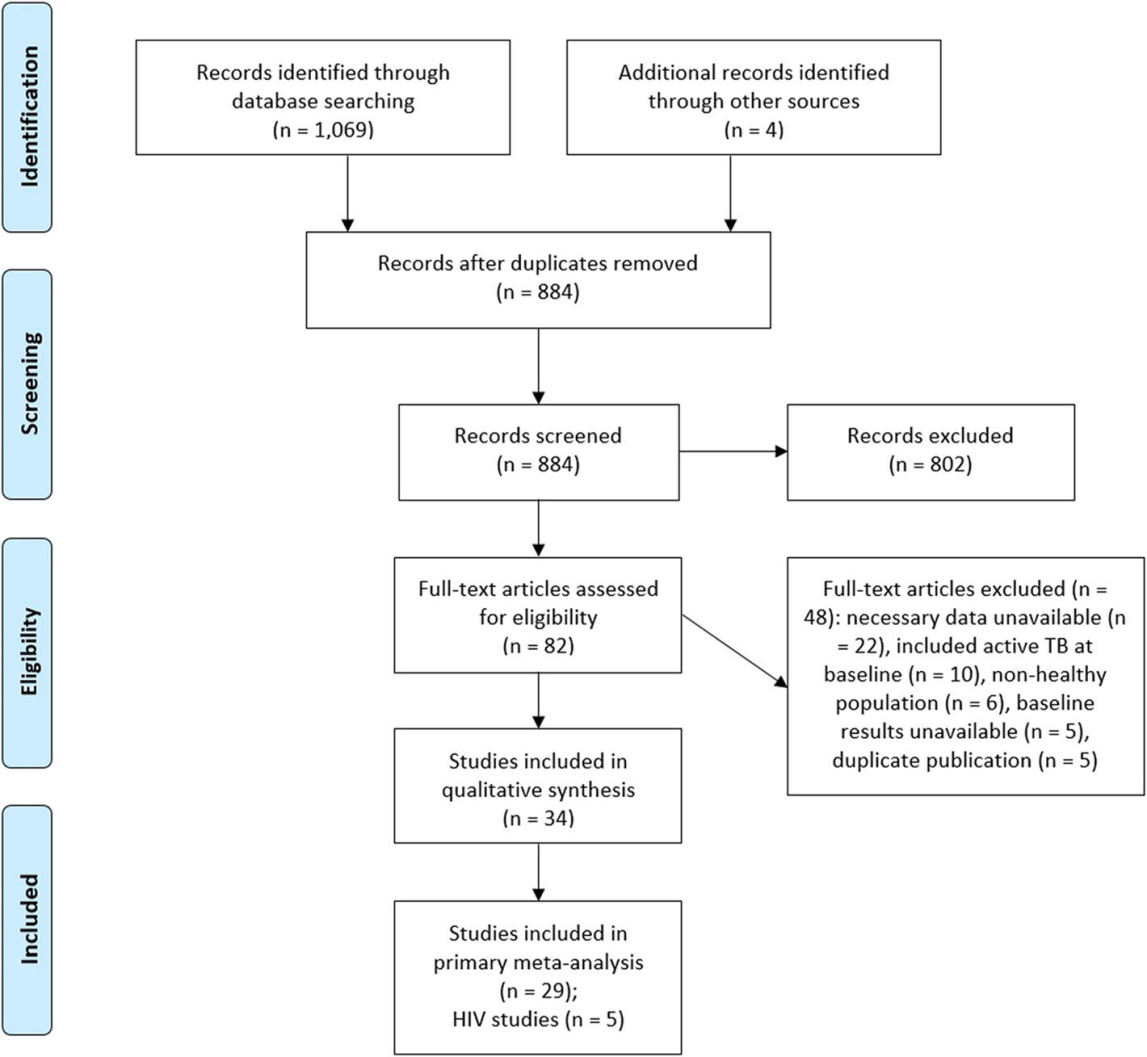
Flow chart of study selection

Our literature search identified a total of 1074 citations (Fig. 1). After removal of duplicate citations, 884 remained for title and abstract review of which 82 were included for full-text assessment. We included 34 studies in our dose-response meta-analysis, 18 of which were conducted in Europe, 8 in Asia, 2 in North America, 2 in the Middle East, 2 in Sub-Saharan Africa, 1 in Latin America, and 1 in Australasia. The 34 studies provided a combined cohort size of 581,956 person-years with a total of 788 incident cases of TB. The median study duration was 2.5 years (IQR 1.75–4.30).

Most study cohorts included participants at elevated risk for TB (Table S1). Of the included studies, 15 cohorts included TB case contacts, 8 cohorts of healthcare workers, and 5 cohorts included migrants or asylum seekers. In addition, 2 studies were population-based and another study included individuals without known exposure to TB. Eight studies provided TPT to participants ranging from 2 to 5% of the study sample with one study providing treatment to 19% of the study sample, while 16 studies did not provide TPT.

### Dose-response relationship

**Fig. 2:**
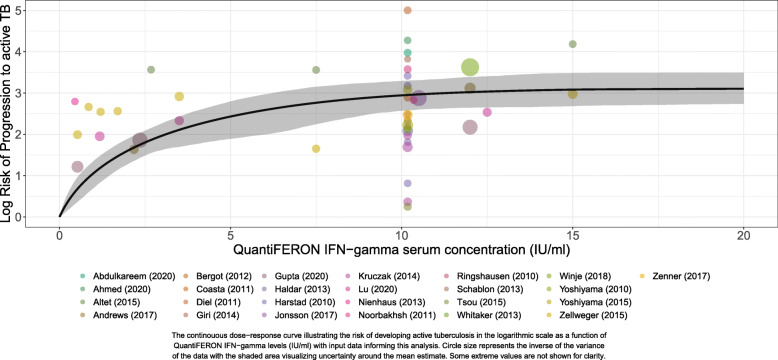
Dose-response curve for the association between interferon gamma levels and risk of developing active tuberculosis

Overall, higher levels of IFN-gamma were associated with increased risk of progression to active tuberculosis (Fig. 2). We find that the risk increased sharply between IFN-gamma levels 0 and 5 IU/ml, after which the risk continued to increase moderately but at a slower pace until reaching about 15 IU/ml where the risk levels off until 20 IU/ml. Table 1 provides quantitative measurements of the dose-response risk curve for the primary analysis. Specifically, the relative risk of progression to active TB compared to 0 IU/ml for those with IFN-gamma levels of 0.20, 0.35, 0.70, 1, 5, 10, 15, and 20 IU/ml were: 1.37 (1.15–1.63), 1.64 (1.28–2.08), 2.31 (1.63–3.09), 2.90 (2.02–3.88), 11.38 (6.64–16.38), 19.00 (13.08–26.90), 21.82 (14.65–32.57), and 22.31 (15.43–33.00), respectively.

**Table 1:** Risk of progression to active TB across IFN-gamma levels (IU/ml) by model

IFN-gamma levels (IU/ml)	Relative Risk (95% Uncertainty interval)					
	Primary Analysis	High Quality Studies^a^	Received TPT	No TPT	Adults	Children
0.00	REF	REF	REF	REF	REF	REF
0.10	1.18 (1.07 to 1.32)	1.23 (1.09 to 1.39)	1.26 (1.05 to 1.52)	1.16 (1.02 to 1.32)	1.24 (1.10 to 1.39)	1.13 (1.00 to 1.41)
0.20	1.37 (1.15 to 1.63)	1.45 (1.19 to 1.77)	1.50 (1.10 to 2.01)	1.32 (1.05 to 1.64)	1.46 (1.21 to 1.76)	1.26 (1.00 to 1.81)
0.30	1.55 (1.23 to 1.93)	1.67 (1.29 to 2.14)	1.74 (1.16 to 2.50)	1.48 (1.10 to 1.94)	1.69 (1.32 to 2.12)	1.37 (1.01 to 2.19)
0.35	1.64 (1.28 to 2.08)	1.78 (1.34 to 2.32)	1.86 (1.19 to 2.74)	1.56 (1.12 to 2.09)	1.80 (1.37 to 2.30)	1.43 (1.01 to 2.38)
0.40	1.74 (1.32 to 2.24)	1.88 (1.39 to 2.50)	1.98 (1.23 to 2.97)	1.64 (1.15 to 2.25)	1.90 (1.42 to 2.47)	1.49 (1.01 to 2.57)
0.50	1.93 (1.42 to 2.54)	2.10 (1.50 to 2.84)	2.22 (1.30 to 3.43)	1.81 (1.22 to 2.54)	2.11 (1.53 to 2.81)	1.60 (1.01 to 2.95)
0.70	2.31 (1.63 to 3.09)	2.52 (1.71 to 3.53)	2.68 (1.47 to 4.33)	2.15 (1.38 to 3.09)	2.52 (1.75 to 3.45)	1.81 (1.02 to 3.67)
1.00	2.90 (2.02 to 3.88)	3.14 (2.10 to 4.45)	3.37 (1.81 to 5.64)	2.67 (1.68 to 3.93)	3.09 (2.10 to 4.32)	2.13 (1.03 to 4.73)
2.00	4.99 (3.61 to 6.65)	5.08 (3.29 to 7.50)	5.59 (3.01 to 9.41)	4.56 (3.03 to 6.52)	4.70 (3.15 to 6.66)	3.10 (1.06 to 7.67)
3.00	7.16 (5.07 to 9.65)	6.80 (4.27 to 10.67)	7.65 (4.01 to 12.38)	6.54 (4.09 to 9.88)	5.89 (3.93 to 8.25)	3.95 (1.15 to 11.06)
4.00	9.32 (5.85 to 13.06)	8.31 (4.69 to 14.30)	9.53 (4.75 to 15.19)	8.50 (4.62 to 13.49)	6.73 (4.16 to 9.57)	4.72 (1.28 to 14.53)
5.00	11.38 (6.64 to 16.38)	9.63 (4.87 to 18.00)	11.26 (5.52 to 18.08)	10.42 (4.85 to 17.51)	7.33 (4.31 to 10.81)	5.48 (1.45 to 18.14)
6.00	13.31 (7.37 to 19.43)	10.83 (5.17 to 21.46)	12.90 (6.33 to 20.97)	12.28 (5.31 to 21.43)	7.80 (4.50 to 12.19)	6.30 (1.72 to 21.91)
7.00	15.07 (8.63 to 22.07)	11.99 (5.67 to 24.60)	14.54 (7.61 to 23.07)	14.11 (6.38 to 24.60)	8.27 (5.01 to 13.19)	7.23 (2.03 to 25.37)
8.00	16.61 (10.31 to 24.16)	13.13 (6.29 to 27.22)	16.17 (9.37 to 25.01)	15.84 (8.08 to 26.98)	8.79 (5.62 to 14.14)	8.25 (2.46 to 27.55)
10.00	19.00 (13.08 to 26.90)	15.16 (7.38 to 31.92)	19.10 (12.87 to 29.37)	18.74 (11.01 to 30.36)	9.85 (6.20 to 15.17)	10.24 (2.96 to 33.94)
12.00	20.53 (14.13 to 29.62)	16.74 (7.90 to 35.79)	21.35 (14.11 to 32.55)	20.75 (12.04 to 34.15)	10.78 (6.79 to 16.66)	11.90 (3.47 to 39.68)
15.00	21.82 (14.65 to 32.57)	18.18 (8.39 to 40.54)	23.26 (14.95 to 37.08)	22.39 (12.88 to 38.79)	11.75 (7.14 to 18.99)	13.44 (4.03 to 45.99)
20.00	22.31 (15.43 to 33.00)	18.74 (8.69 to 41.61)	23.82 (15.26 to 37.92)	22.94 (13.27 to 39.56)	12.22 (7.43 to 19.48)	14.04 (4.33 to 46.61)

^a^Sensitivity analysis excluding studies with NOS score < 5

NOTE: TPT (TB preventive treatment). In the TPT subgroup analyses, data inputs into the models were stratified by whether studies provided any TPT. The children subgroup analysis included data for individuals below 18 years, while the adult subgroup analysis included data for those 18 years and older

### Sensitivity analyses

**Fig. 3:**
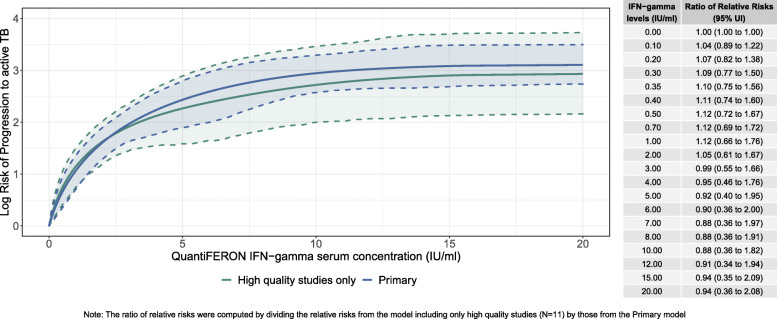
Sensitivity analysis results (excluding studies with NOS quality score < 5)

The quality assessment scores for each study are in Table S1. According to the quality assessment, the quality of the including studies ranged between 6, indicating moderate quality, and 3, indicating lower quality. Particularly, 14 (41%) studies scored between 5 and 6, 12 (35%) studies scored a 4, and the remaining 8 (24%) studies scored a 3. Sensitivity analyses that excluded studies with an NOS score lower than 5 exhibit similar results to models utilizing the complete dataset (Fig. 3; Table 1). While the curve including high-quality studies was steeper at the lower end and lower at the higher end compared to the primary analysis, the differences were not statistically significant.

### Subgroup analyses

**Fig. 4:**
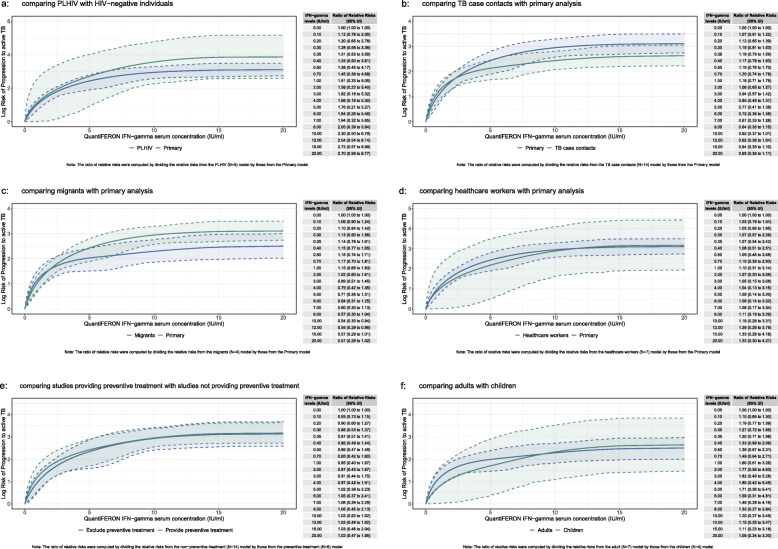
Subgroup analysis results: **a** comparing PLHIV with HIV-negative individuals, **b** comparing TB case contacts with primary analysis, **c** comparing migrants with primary analysis, **d** comparing healthcare workers with primary analysis, **e** comparing studies providing preventive treatment with studies not providing preventive treatment, **f** comparing adults with children

In subgroup analyses, there was some heterogeneity in risk across IFN-gamma levels for at risk populations. The risk of TB among people living with HIV (PLHIV) was higher compared to the rate found in the primary analysis. Across all levels of IFN-gamma, the dose-response risk curve for PLHIV was substantially higher compared to the primary analysis (Fig. 4a; Table 2). Among TB case contacts (Fig. 4b; Table 2) and migrants (Fig. 4c; Table 2) the risk was steeper at the lower end of IFN-gamma levels (< 2.5 IU/ml) but the risk was lower in the remaining IFN-gamma levels compared to the primary analysis. For healthcare workers (Fig. 4d; Table 2), the dose-response risk curve yielded similar results to the primary analysis. Similarly, we found no difference in the risk of progression to active TB when stratifying results by whether studies provided TPT. (Fig. 4e; Table 1). Finally, we found that the dose-response risk curve was steeper at the lower end (< 7.5 IU/ml) for adults compared to children but the risks converged afterwards (Fig. 4f; Table 1). Additional subgroup analyses on time to incident TB, definition of active TB, and TB incidence of country of study showed no significant differences (Fig. S1-S3).

**Table 2:** Risk of progression to active TB across IFN-gamma levels (IU/ml) by risk group

IFN-gamma levels (IU/ml)	Relative Risk (95% Uncertainty interval)			
	People living with HIV	TB Case Contacts	Healthcare workers	Migrants
0.00	REF	REF	REF	REF
0.10	1.28 (1.00 to 2.33)	1.26 (1.12 to 1.40)	1.20 (1.00 to 1.79)	1.25 (1.11 to 1.42)
0.20	1.51 (1.00 to 3.62)	1.51 (1.24 to 1.78)	1.37 (1.00 to 2.57)	1.50 (1.22 to 1.84)
0.30	1.73 (1.00 to 4.88)	1.76 (1.37 to 2.16)	1.54 (1.00 to 3.35)	1.73 (1.33 to 2.24)
0.35	1.83 (1.00 to 5.50)	1.88 (1.43 to 2.34)	1.62 (1.00 to 3.74)	1.85 (1.39 to 2.43)
0.40	1.94 (1.01 to 6.11)	2.00 (1.49 to 2.52)	1.70 (1.01 to 4.12)	1.96 (1.44 to 2.62)
0.50	2.14 (1.01 to 7.36)	2.23 (1.62 to 2.87)	1.85 (1.01 to 4.89)	2.19 (1.56 to 3.00)
0.70	2.56 (1.01 to 9.85)	2.69 (1.89 to 3.54)	2.16 (1.02 to 6.39)	2.62 (1.78 to 3.71)
1.00	3.21(1.02 to 13.27)	3.33 (2.29 to 4.46)	2.61 (1.02 to 8.34)	3.23 (2.12 to 4.68)
2.00	5.62 (1.10 to 24.32)	5.17 (3.62 to 6.92)	4.15 (1.05 to 14.45)	4.93 (3.32 to 7.27)
3.00	8.39 (1.35 to 34.57)	6.58 (4.61 to 8.77)	5.69 (1.16 to 19.84)	6.16 (4.18 to 8.95)
4.00	11.50 (1.73 to 45.48)	7.62 (5.01 to 10.29)	7.24 (1.33 to 26.21)	7.02 (4.43 to 10.26)
5.00	14.99 (2.49 to 55.66)	8.39 (5.28 to 11.66)	8.84 (1.65 to 32.72)	7.61 (4.53 to 11.10)
6.00	18.83 (3.56 to 67.91)	9.01 (5.47 to 13.16)	10.53 (2.09 to 38.31)	8.06 (4.62 to 12.33)
7.00	23.00 (5.07 to 78.98)	9.60 (5.95 to 14.44)	12.35 (2.75 to 43.89)	8.49 (4.96 to 13.43)
8.00	27.30 (6.68 to 94.05)	10.21 (6.77 to 15.17)	14.26 (3.56 to 48.25)	8.97 (5.58 to 14.43)
10.00	35.34 (9.59 to 119.43)	11.37 (7.98 to 16.53)	17.84 (5.06 to 60.08)	9.94 (6.44 to 15.71)
12.00	41.74 (11.25 to 146.63)	12.33 (8.49 to 18.17)	20.73 (5.87 to 72.65)	10.79 (6.81 to 17.48)
15.00	46.86 (13.17 to 173.69)	13.35 (9.01 to 20.46)	23.20 (6.66 to 82.24)	11.72 (7.17 to 19.42)
20.00	47.70 (13.46 to 175.51)	13.84 (9.24 to 21.09)	23.85 (7.02 to 83.46)	12.19 (7.57 to 20.11)

## Discussion

To our knowledge, this is the first meta-analysis to summarize and quantify the dose-response relationship between QuantiFERON-TB IFN-gamma levels and the risk of progression to active TB using all available global data sources. Using data from 34 studies, we found that the risk of TB development increased with higher IFN-gamma levels. Our continuous dose-response curve indicates that the risk of incident TB sharply increases between IFN-gamma levels of 0 and 5 IU/ml after which the risk continued to increase moderately but at a slower pace until reaching 15 IU/ml where the risk levels off. Sensitivity analyses revealed that our findings are robust to the quality of the studies as the results did not differ significantly by the quality of studies.

We found that the risk of TB is higher with larger IFN-gamma levels. Our findings show that the risk for incident TB is 2.90-fold higher at 1 IU/ml, 10.38-fold higher at 5 IU/ml, 19.00-fold higher at 10 IU/ml, 21.82-fold higher at 15 IU/ml, and 22.31-fold higher at 20 IU/ml. These findings underscore a limitation of the current practice of dichotomizing IGRA results where the interpretation is that risk is constant for all positive IGRA test. However, our findings show that the risk of active TB development is not the same for everyone with a positive IGRA when considering the full spectrum of positive IGRA reactions. In addition, the results indicate that very high IFN-gamma levels from a QuantiFERON-TB test, beyond the traditional threshold, are powerful indicators of progression to active TB from latent infection. Some authors have suggested that these very high levels of IFN-gamma levels are markers for subclinical active TB disease or early incipient disease [12, 18].

The dose-response relationship found in this meta-analysis can be used to help guide clinical decisions to perform additional tests and treat latent tuberculosis infection within the context of TB programs and local epidemiology. Guidance in clinical decisions would particularly be useful in high TB burden areas where preventive treatment for latent TB is under-utilized [19, 20]. The results from this meta-analysis can help make clinical decisions more efficient when results are in the intermediate area between a negative and positive IGRA test called the borderline zone. A recent large cohort study found that half of the patients with an IFN-gamma level in the borderline zone (0.20–1.00 IU/ml) were IGRA negative in a follow-up test [14]. Results in this borderline zone can provide justification for secondary IGRA tests to prevent unnecessary treatment. Furthermore, our results show that the risk of incident TB is markedly greater in people with high IFN-gamma levels. Current WHO recommendations are to provide preventive treatment to a subset of risk groups such as PLHIV and TB case contacts [8]. The findings from this study suggest an opportunity to consider the potential benefit of extending TB preventive treatment to those with high IFN-gamma levels while taking into account risk factors for disease progression.

We found some variation in the dose-response relationship by population. Compared to our primary curve that excluded studies where the entire study population is HIV positive, the dose-response relationship was substantially higher in studies among HIV positive individuals. Though some studies have found that IGRAs have modest predictive power for incident TB among HIV positive individuals [21, 22], our results are consistent with studies indicating that IGRAs are sensitive tools for predicting TB progression among PLHIV [23–25].

In addition, we found some differences in the dose-response relationship when stratifying analyses by adults and children. The relative risk of incident TB was lower for children compared to adults at the lower end of the curve before converging at 7.5 IU/ml. The different risk is likely due to mixed findings on the utility of IGRA’s among very young children. Several studies have found that there is insufficient reaction to *M. tuberculosis* antigen among young children that adversely impacts IGRA results [26–28]. More investigations are needed to assess the efficacy of IGRAs to predict progression to active TB to confirm the performance of IGRA at lower levels of IFN-gamma levels.

Contrary to expectations, we found no evidence of effect modification when stratifying models by whether studies provided preventive treatment for TB. The lack of difference in the curves can be due to the fact that only a small proportion of participants accepted treatment in studies where it was provided. In addition, even a smaller fraction of those accepting treatment completed the regimen in these studies.

Finally, the findings from this study bring into question the predictive value for progression to active TB of IGRAs. Various systematic reviews have concluded that IGRAs have poor accuracy for the prediction of incident TB [21, 29]. These results may change by taking into account the full dynamic nature of latent infection instead of dichotomizing IGRA results with conventional thresholds. In studies where IFN-gamma levels were evaluated at larger cutoffs beside the threshold of 0.35 IU/ml, the predictive value of subsequent TB improved [10, 12, 30, 31].

### Strengths and limitations

Our study has several key strengths. In our meta-regression we were able to include data with different IFN-gamma categories into a singular analysis while incorporating between study heterogeneity in our uncertainty estimation. This is an advantage over traditional methods that would have to take the midpoint of IFN-gamma categories rather than use information of the entire category as done in this study. Our study is the first meta-analysis to examine TB risk over the entire distribution of IFN-gamma levels, allowing for improved identification of individuals who may be at highest risk for progressing to active TB. Finally, we were able to stratify results by important at risk populations to evaluate for potential confounding and effect modification.

Our findings should be interpreted in terms of key limitations. First, we could not assess for effect modification by known risk factors for progression to active TB including tobacco smoke, alcohol consumption, diabetes, malnutrition, and indoor air pollution, as these data were not routinely included in studies. Second, several studies included in our systematic review used passive-follow-up for detection of active TB through national TB registries. These surveillance systems are often prone to under-reporting which may have caused lower rates of observed TB cases in the studies. Third, our quality assessment indicated that almost all studies have some source of bias as all studies were considered to be of low to moderate quality. We conducted sensitivity analyses to evaluate the impact of study quality on the results, and we found that the results did not differ significantly. Fourth, most studies were conducted in low to intermediate TB burden countries, potentially limiting the generalizability of our findings. Fifth, time since infection may impact results as recent infection is associated with higher risk for active TB. However, this information was unavailable in most studies. Finally, we may have missed articles as we restricted our search to two databases. We believe this had a small impact on our findings because PubMed and Embase yield high coverage [32] and we manually searched the reference list of relevant articles.

Our study has implications for future studies. We found that the risk of incident TB is not the same for everyone with a positive IGRA reaction. Future cohort studies should therefore collect granular data on IGRA levels and the associated risk of progression to active tuberculosis. For example, out of the 34 included studies in our systematic review, only 9 reported more IGRA values besides the traditional cutoff of 0.35 IU/ml. Reporting more granular IGRA values and corresponding risks for progression to active TB will also help reassess the predictive value of IGRAs given the dose-response nature of the data. Future studies may also incorporate additional risk factors for progression to active tuberculosis such as alcohol, smoking, malnutrition, and diabetes to identify individuals at greatest risk of subsequent TB.

## Conclusion

We developed a dose-response risk curve for the progression to active TB as a function of a continuous measure of IGRA values. Our findings show that the current practice of dichotomizing IGRA test results simplifies the TB infection disease continuum. The findings of this study showed that the risk of active TB development is not the same for everyone with a positive IGRA, with higher IGRA values being strongly associated with disease progression. With IGRAs starting to scale up in high TB burden areas, the findings from this study can help clinicians make informed decisions by providing different relative risks of progression to active TB for a range of IGRA values within the borderline zone and very high IGRA reactions. More investigations using detailed quantification of IGRA values will help to find more accurate estimates of the dose-response relationship and allow for a reexamination of the predictive power of IGRA tests.

## Supplementary Information

**Additional file 1: Supplementary file S1**: Data Supplement. **Table S1**. Characteristics of the studies included in the meta-analysis. **Table S2**. Search strategies. **Table S3**. Newcastle-Ottawa quality assessment scale adopted for quality assessment. **Figure S1**. Sensitivity analysis results (comparing time to incident TB of 2 or 3 months to 6 months or more). **Figure S2**. Sensitivity analysis results (comparing bacteriologically confirmed TB to clinically confirmed TB). **Figure S3**. Sensitivity analysis results (comparing all form TB incidence in country of study by < 40 per 100,000 and > 40 per 100,000). **Figure S4**. Sensitivity analysis results (excluding studies that provided TB preventive treatment)**.** PRISMA-P 2015 Checklist.

## Data Availability

The data sets used and/or analyzed during the current study are available from the corresponding author on reasonable request.

## Abbreviations

TB: Tuberculosis
IGRA: Interferon-gamma release assay
PRISMA: Preferred Reporting Items for Systematic Review
MOOSE: Meta-analysis of Observational Studies in Epidemiology
LTBI: Latent Tuberculosis Infection
TST: Tuberculin Skin Test
QFT-GIT: QuantiFERON TB Gold in Tube
WHO: World Health Organization
NOS: Newcastle-Ottawa Quality Assessment Scale
PLHIV: People living with HIV
TPT: Tuberculosis Preventive Treatment
