# Supplementary material for: Interferon-gamma release assay levels and risk of progression to active tuberculosis: a systematic review and dose-response meta-regression analysis
Source: BMC Infect Dis. 2021 May 22;21:467. doi: 10.1186/s12879-021-06141-4 (PMC8141158; doi:10.1186/s12879-021-06141-4)
Supplement: Supplementary file 1 — Additional file 1: Supplementary file S1: Data Supplement. Table S1. Characteristics of the studies included in the meta-analysis. Table S2. Search strategies. Table S3. Newcastle-Ottawa quality assessment scale adopted for quality assessment. Figure S1. Sensitivity analysis results (comparing time to incident TB of 2 or 3 months to 6 months or more). Figure S2. Sensitivity analysis results (comparing bacteriologically confirmed TB to clinically confirmed TB). Figure S3. Sensitivity analysis results (comparing all form TB incidence in country of study by < 40 per 100,000 and > 40 per 100,000). Figure S4. Sensitivity analysis results (excluding studies that provided TB preventive treatment). PRISMA-P 2015 Checklist. [file 12879_2021_6141_MOESM1_ESM.docx]

**Supplementary Appendix**

Interferon-gamma release assay levels and risk of progression to active tuberculosis: a systematic review and dose-response meta-regression analysis

Table of Contents

**Table S1. Characteristics of the studies included in the meta-analysis1**

**Table S2. Search strategies** **6**

**Table S3. Newcastle-Ottawa quality assessment scale adopted for quality assessment** **7**

**Figure S1. Sensitivity analysis results (comparing time to incident TB of 2 or 3 months to 6 months or more)** **8**

**Figure S2. Sensitivity analysis results (comparing bacteriologically confirmed TB to clinically confirmed TB)** **8**

**Figure S3. Sensitivity analysis results (comparing all form TB incidence in country of study by < 40 per 100,000 and > 40 per 100,000 )** **9**

**Figure S4. Sensitivity analysis results (excluding studies that provided TB preventive treatment)** **9**

**Estimating the relative risks of developing active TB by interferon gamma levels** **10**

**PRISMA-P 2015 Checklist** **11**

**Table S1. Characteristics of the studies included in the meta-analysis**

| **Study** | **Study Type** | **Population** | **Country** | **Time period** | **Age** | **Sample size** | **Follow-up time** | **Time to incident TB case** | **TB Preventive Treatment** | **TB diagnosis** | **Incident TB cases** | **NOS Quality Score** |
| --- | --- | --- | --- | --- | --- | --- | --- | --- | --- | --- | --- | --- |
| Abdulkareem (2020)(1) | Prospective cohort | Tuberculosis case contacts | Iraq | 2018 – 2018 | Adults and children  1 to 90 years | 401 | 6 months | 3 to 6 Months | Unknown | Bacteriologically, radiologically | 0–0.35 IU/ml: 0/323  0.35–20 IU/ml: 6/78 | 4 |
| Ahmed (2020)(2) | Prospective cohort | High risk population: case contacts, recent migrants | United States of America | 2012 – 2018 | Children 0 to 15 years | 3593 | Median: 4.3 Years | 2 to 24 Months  (2 cases in 2 months) | No 0 % | Bacteriologically | 0–0.35 IU/ml: 1/3238  0.35–20 IU/ml: 3/355 | 6 |
| Aichelburg (2009)(3) | Prospective cohort | HIV-infected patients | Austria | 2006 – 2008 | Adults and children  0 to 100 years | 775 | Median: 1.6 Years | Median: 287 days | No 0 % | Clinically | 0–0.35 IU/ml: 0/738  0.35–20 IU/ml: 3/37 | 6 |
| Altet (2015)(4) | Prospective cohort | Tuberculosis case contacts | Spain | 2007 – 2013 | Adults and children  0 to 100 years | 937 | 4 Years | 6 to 24 Months | No 0 % | Clinically confirmed through TB Control Program databases | 0–0.35 IU/ml: 0/531  0.35–5 IU/ml: 4/135  5–10 IU/ml: 5/166  10–20 IU/ml: 6/105 | 5 |
| Andrews (2017)(5)* | Double blinded, randomized clinical trial | Young healthy children | South Africa | 2009 – 2012 | Children 4 to 6 months | 2374 | 2 Years | 11 to 58 Months | Yes unknown % | Bacteriologically | 0–0.35 IU/ml: 11/2232  0.35–4 IU/ml: 2/79  4–20 IU/ml: 7/63 | 4 |
| Bergot (2012)(6) | Prospective cohort | Tuberculosis case contacts | France | 2007 – 2009 | Adults and children  11 to 97 years | 674 | Mean: 2.8 Years | 17 to 18 Months | No 0 % | Clinically, radiologically, bacteriologically, histology | 0–0.35 IU/ml: 1/526  0.35–20 IU/ml: 1/148 | 6 |
| Costa (2011)(7) | Prospective cohort | Healthcare workers | Portugal | 2007 – 2010 | Adults 18 to 100 years | 2865 | Mean: 1.6 Years | 4 to 24 Months | Unknown | Radiologically | 0–0.35 IU/ml: 0/1908  0.35–20 IU/ml: 4/957 | 3 |
| Diel (2011)(8) | Prospective cohort | Tuberculosis case contacts | Germany | 2005 – 2010 | Adults and children  0 to 100 years | 954 | Mean: 2.5 Years | 3 to 23 Months | No  0% | Bacteriologically, radiologically, clinically | 0–0.35 IU/ml: 0/756  0.35–20 IU/ml: 19/198 | 4 |
| Doyle (2014)(9) | Retrospective cohort | HIV-infected patients | Australia | 2003 – 2011 | Adults  18 to 88 years | 913 | Median: 2.2 Years | within 24 months | Provided:  Yes – 2 %  Completed:  <1 % | Bacteriologically | 0–0.35 IU/ml: 1/884  0.35–20 IU/ml: 1/29 | 4 |
| Giri (2014)(10) | Retrospective cohort | Healthcare workers | United Kingdom | 2009 – 2013 | Adults and children  0 to 100 years | 1258 | 1 Year | Not reported | Provided:  Yes – 4 %  Completed:  3 % | Bacteriologically, clinically | 0–0.35 IU/ml: 0/1162  0.35–20 IU/ml: 0/96 | 4 |
| Gupta (2020)(11) | Prospective cohort | High risk population: case contacts, migrants | United Kingdom | 2010 – 2017 | Adults 16 to 100 years | 8440 | Median: 4.7 Years | Median: 188 days | No 0 % | Clinically confirmed through national TB surveillance program | 0–0.35 IU/ml: 34/6637  0.35–5 IU/ml: 7/405  5–10 IU/ml: 27/820  10–20 IU/ml: 26/578 | 5 |
| Haldar (2013)(12) | Prospective cohort | Tuberculosis case contacts | United Kingdom | 2007 – 2009 | Adults 16 to 100 years | 811 | Median: 2.5 Years | Median: 171 days | No 0 % | Bacteriologically, clinically | 0–0.35 IU/ml: 6/601  0.35–20 IU/ml: 14/210 | 5 |
| Harstad (2010)(13) | Prospective cohort | Asylum seekers | Norway | 2005 – 2008 | Adults 18 to 100 years | 823 | 23 to 32 months | 2 to 20 Months  (1 case in 2 months) | No  0% | Clinically confirmed through National TB registry | 0–0.35 IU/ml: 0/577  0.35–20 IU/ml: 6/238 | 3 |
| Jonsson (2017)(14) | Retrospective cohort | All residents meeting Swedish guidelines for LTBI testing | Sweden | 2009 – 2016 | Adults and children  0 to 100 years | 38785 | Median: 4.3 Years | 3 to 24+ Months | Yes  unknown % | Clinically confirmed through national TB register | 0–0.35 IU/ml: 23/30567  0.35–1 IU/ml: 15/1937  1–20 IU/ml: 84/6281 | 3 |
| Joshi (2011)(15) | Prospective cohort | Healthcare workers | India | 2004 – 2010 | Adults 18 to 100 years | 726 | 6 Years | 3+ Months | Yes  unknown % | Bacteriologically, clinically | 0–0.35 IU/ml: 8/2365  0.35–20 IU/ml: 6/1626 | 4 |
| Kruczak (2014)(16) | Prospective cohort | High risk population: homeless, contacts, elderly, healthy group | Poland | 2007 – 2012 | Adults and children  0 to 100 years | 785 | 4 to 5 Years | 6 to 20 Months | No 0 % | Clinically confirmed through local pulmonary clinic | 0–0.35 IU/ml: 4/574  0.35–20 IU/ml: 8/211 | 5 |
| Lee (2019)(17) | Retrospective cohort | HIV-infected patients | Republic of Korea | 2006 – 2016 | Adults 15 to 100 years | 416 | Median: 4 Years | 3+ Months | Provided:  Yes – 5 %  Completed:  unknown % | Bacteriologically, radiologically | 0–0.35 IU/ml: 1/354  0.35–20 IU/ml: 4/62 | 5 |
| Lu (2020)(18) | Prospective cohort | Population based | China | 2013 – 2019 | Adults and children  5 to 100 years | 5,405 | 6 Years | 3+ Months | Unknown | Bacteriologically, clinically | 0–0.35 IU/ml: 4/25806  0.35 – 20 IU/ml: 9/6624 | 5 |
| Mahomed (2011)(19) | Prospective cohort | Children | South Africa | 2005 – 2009 | Children 12 to 18 years | 5244 | Median: 2.4 Years | 6+ Months | No 0 % | Bacteriologically | 0–0.35 IU/ml: 13/2575  0.35–20 IU/ml: 39/2669 | 5 |
| Nienhaus (2013)(20) | Prospective cohort | Healthcare workers | Portugal | 2007 – 2010 | Adults and children  0 to 100 years | 2815 | Mean: 1.1 Years | 4 to 24 Months | Unknown | Clinically, radiologically, symptoms | 0–0.20 IU/ml: 0/1764  0.20–0.70 IU/ml: 1/323  0.70–20 IU/ml: 3/728 | 3 |
| Noorbakhsh (2011)(21) | Prospective cohort | Tuberculosis case contacts | Iran | 2006 – 2008 | Children 0 to 19 years | 49 | 1 Year | 3+ Months | Unknown | Clinically | 0–0.35 IU/ml: 0/31  0.35–20 IU/ml: 10/18 | 4 |
| Ringshausen (2010)(22) | Prospective cohort | Healthcare workers | Germany | 2005 – 2010 | Adults 19 to 62 years | 180 | Median: 2.7 years | Not reported | Unknown | Radiologically | 0–0.35 IU/ml: 0/162  0.35–20 IU/ml: 0/18 | 3 |
| Rose (2014)(23) | Retrospective cohort | HIV-infected patients | Canada | 2010 – 2011 | Children 0 to 18 years | 80 | 2 year | Not reported | Provided:  Yes – 5 %  Completed:  unknown % | Bacteriologically, clinically | 0–0.20 IU/ml: 0/65  0.20–0.70 IU/ml: 0/9  0.70–20 IU/ml: 0/6 | 3 |
| Santin (2011)(24) | Prospective cohort | HIV-infected patients | Spain | 2007 – 2009 | Adults 18 to 100 years | 135 | Median: 1.6 Years | Not reported | No 0 % | Clinically confirmed through national TB surveillance program | 0–0.35 IU/ml: 0/122  0.35–20 IU/ml: 0/13 | 4 |
| Schablon (2013)(25) | Prospective cohort | Healthcare workers | Germany | 2008 – 2012 | Adults 17 to 53 years | 194 | 3 years | Not reported | Unknown | Clinically, radiologically | 0–0.35 IU/ml: 0/190  0.35–20 IU/ml: 0/4 | 4 |
| Sharma (2017)(26) | Prospective cohort | Tuberculosis case contacts | India | 2008 – 2014 | Adults and children  1 to 65 years | 1498 | 2 Years | 11 to 15 Months | Unknown | Bacteriologically, clinically | 0–0.35 IU/ml: 19/581  0.35–20 IU/ml: 56/917 | 4 |
| Tsou (2015)(27) | Prospective cohort | Elderly nursing home residents | Taiwan (Province of China) | 2004 – 2009 | Adults 65 to 100 years | 139 | 5 years | 4 to 5 Years | Unknown | Clinically | 0–0.35 IU/ml: 2/100  0.35–20 IU/ml: 1/39 | 5 |
| Verhagen (2014)(28) | Prospective cohort | Tuberculosis case contacts | Venezuela (Bolivarian Republic of) | 2010 – 2012 | Children 0 to 15 years | 140 | 1 Year | 6 to 12 Months | No 0 % | Clinically confirmed through national TB surveillance program | 0–0.35 IU/ml: 2/77  0.35–20 IU/ml: 2/63 | 5 |
| Whitaker (2013)(29) | Prospective cohort | Healthcare workers | Georgia | 2009 – 2011 | Adults 18 to 100 years | 319 | 2.2 Years | 12 Months | Unknown | Clinically confirmed through TB Control Program databases | 0–0.35 IU/ml: 0/173  0.35–20 IU/ml: 3/146 | 3 |
| Winje (2018)(30) ** | Prospective cohort | Population based | Norway | 2009 – 2014 | Adults and children  0 to 100 years | 44006 | 2 to 8.5 years | Median: 9 Months | No 0 % | Clinically confirmed through national TB surveillance program | 0–0.35 IU/ml: 15/22077  0.35–1 IU/ml: 14/2020  1–4 IU/ml: 48/2553  4–20 IU/ml: 143/4893 | 5 |
| Yoshiyama (2010)(31) | Retrospective cohort | Tuberculosis case contacts | Japan | 2003 – 2007 | Adults and children  10 to 100 years | 3102 | Mean: 1.6 Years | 3+ Months | No 0 % | Bacteriologically, confirmed through public health centers | 0–0.35 IU/ml: 19/2683  0.35–20 IU/ml: 20/419 | 4 |
| Yoshiyama (2015)(32) | Retrospective cohort | Tuberculosis case contacts | Japan | 2010 – 2013 | Adults and children  0 to 100 years | 625 | Mean: 1.7 Years | 3+ Months | No 0 % | Bacteriologically, clinically | 0–0.35 IU/ml: 2/547  0.35–20 IU/ml: 10/168 | 4 |
| Zellweger (2015)(33) | Prospective cohort | Tuberculosis case contacts | Europe | 2009 – 2013 | Adults and children  0 to 100 years | 3425 | Median: 2.5 | 84 to 968 Days | Provided:  Yes – 19 %  Completed:  Unknown % | Clinically confirmed through national TB surveillance program | 0–0.35 IU/ml: 3/2410  0.35–0.70 IU/ml: 2/219  0.70–1 IU/ml: 1/56  1–1.40 IU/ml: 1/63  1.40–2 IU/ml: 1/62  2-5 IU/ml: 4/74  5-10 IU/ml: 1/154  10-20 IU/ml: 7/287 | 3 |
| Zenner (2017)(34) | Retrospective cohort | Migrants | United Kingdom | 2009 – 2014 | Adults and children  0 to 35 years | 1320 | Median: 2.2 | Median: 1 year | No 0 % | Clinically confirmed through national TB register | 0–0.35 IU/ml: 4/971  0.35–20 IU/ml: 9/349 | 5 |

Note: Assumed lower and upper age bound of 0 and 100 if study did not report the range of ages

* Study participants were from a clinical trial assessing the efficacy of MVA85A vaccine for preventing tuberculosis. Results showed that the though the vaccine was safe, it was ultimately ineffective in reducing the risk of developing tuberculosis.(35)

** Only extracted data among healthy individuals

**Table S2. Search strategies**

| **Study type** | **Search Terms** |
| --- | --- |
| Retrospective or prospective cohort studies | PubMed search terms: (“Interferon-gamma Release Tests”[MeSH] OR “IGRA”[tiab] OR "interferon-gamma release assay*"[tiab] OR "Quantiferon*"[tiab] OR "QFT"[tiab] OR "Interferon-gamma release test*"[tiab]) AND ("reactivation"[tiab] OR "reactivity"[tiab] OR "activation"[tiab] OR "predictive"[tiab] OR "risk"[tiab]) AND ("tuberculosis"[MeSH] OR "tuberculosis"[tiab]) AND ("prospective"[tiab] OR "cohort"[tiab] OR "follow up"[tiab]) (“2001/01/01”[PDAT] : "2020/05/10"[PDAT])  Embase search terms: (‘Interferon-gamma Release Tests’/de OR ‘IGRA’:ab,ti OR ‘interferon-gamma release assay*’:ab,ti OR ‘Quantiferon*’:ab,ti OR ‘QFT’:ab,ti OR ‘Interferon-gamma release test*’:ab,ti) AND (‘reactivation’:ab,ti OR ‘reactivity’:ab,ti OR ‘activation’:ab,ti OR ‘predictive’:ab,ti OR ‘risk’:ab,ti) AND (‘tuberculosis’/de OR ‘tuberculosis’:ab,ti) AND (‘prospective’:ab,ti OR ‘cohort’:ab,ti OR ‘follow up’:ab,ti) AND [1-1-2001]/sd NOT [5-10-2020]/sd |

**Table S3. Newcastle-Ottawa quality assessment scale adopted for quality assessment**

| **Criteria Number** | **Criteria Domain** | **Criteria** | **Score** |
| --- | --- | --- | --- |
| 1 | Cohort selection | Representativeness of the exposed cohort | Truly or somewhat representative of the community (*) Selected groups No clear description |
| 2 | Cohort selection | Selection of the non-exposed cohort | Drawn from the same community as exposed cohort (*) Drawn from a different source No clear description |
| 3 | Cohort selection | Ascertainment of exposure | Secured records or structured interviews (*) Written self-report No clear description |
| 4 | Cohort selection | Demonstration that focal outcome was not present at start of study | Excluded participants with baseline TB (*) No |
| 5 | Comparability  of cohort | Comparability: study excluded participants with preventative treatment for LTBI | Yes (*) No |
| 6 | Comparability  of cohort | Comparability: study excluded participants with HIV-infection | Yes (*) No |
| 7 | Outcome | Assessment of outcome | All TB cases were bacteriologically confirmed (*) Clinically diagnosed No clear description |
| 8 | Outcome | Sufficient follow-up for outcome to occur | 3 years > (*) < 3 years No clear description |
| 9 | Outcome | Adequate follow-up of cohort | Loss to follow-up < 20% (*) Loss to follow-up > 20% No clear description |
| Note: For studies where the study population was HIV-positive individuals criteria number 6 was not considered instead studies would be awarded 2 points for criteria number 5 if requirements are met.  Answers with a (*) indicate that study quality improved, while answers without the (*) indicate no improvement in quality. | | | |

**Figure S1: Sensitivity analysis results (comparing time to incident TB of 2 or 3 months (N=9) to 6 months or more (N=11))**


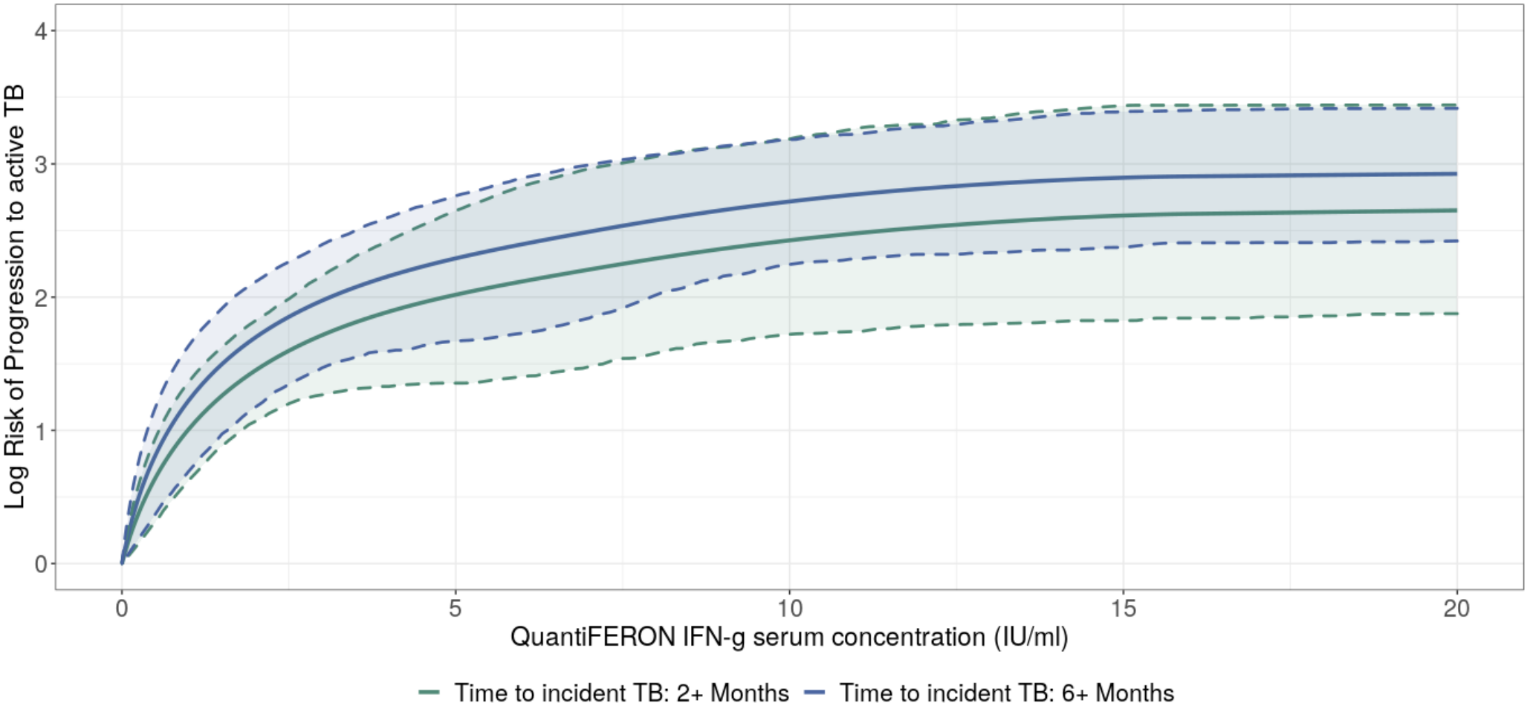


Note: Studies were stratified by whether the first incident TB case was between 2 and 3 months or 6 months

**Figure S2: Sensitivity analysis results (comparing bacteriologically confirmed TB (N=10) to clinically confirmed TB (N=11))**


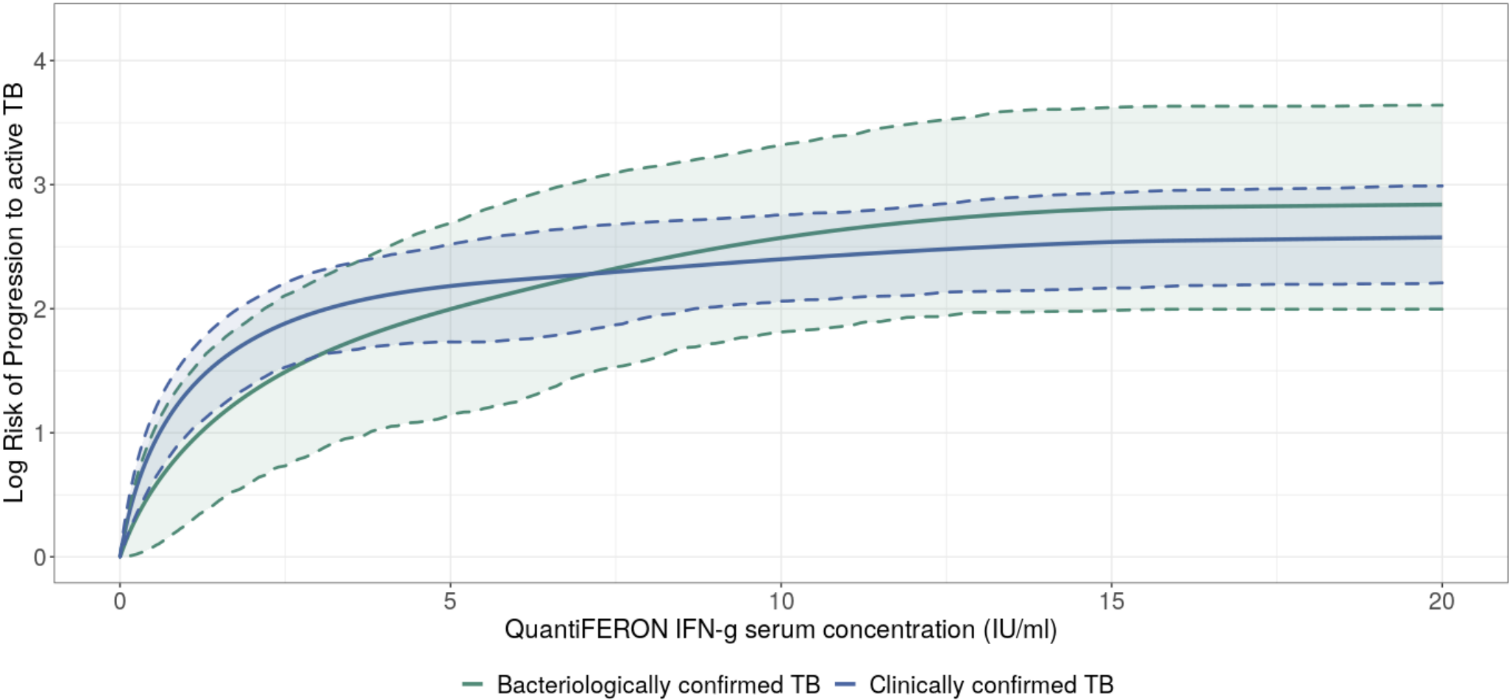


**Figure S3: Sensitivity analysis results (comparing TB incidence in country of study of < 40 per 100,000 and > 40 per 100,000)**

**Figure S3: Sensitivity analysis results (comparing all form TB incidence in country of study by < 40 per 100,000 (N=23) and > 40 per 100,000 (N=6))**


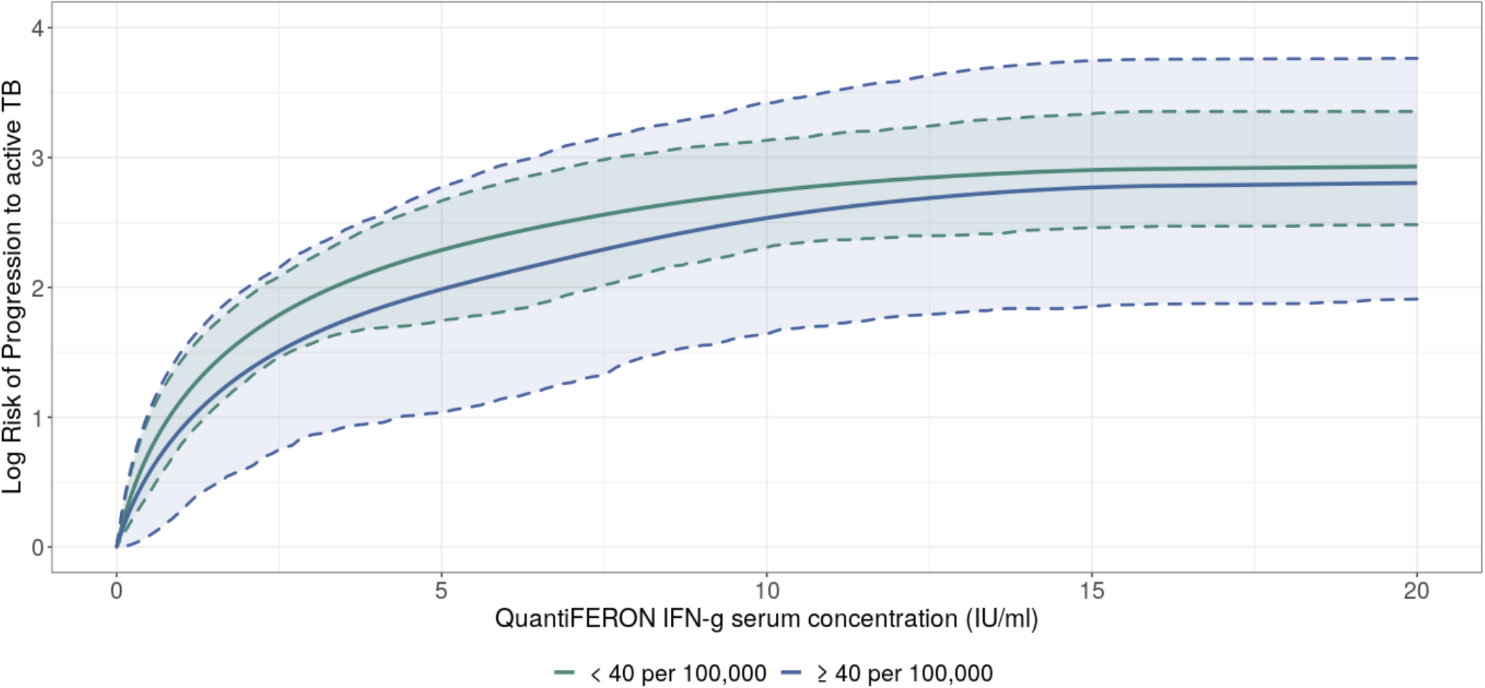


**Figure S4: Sensitivity analysis results (excluding studies that provided TB preventive treatment)**


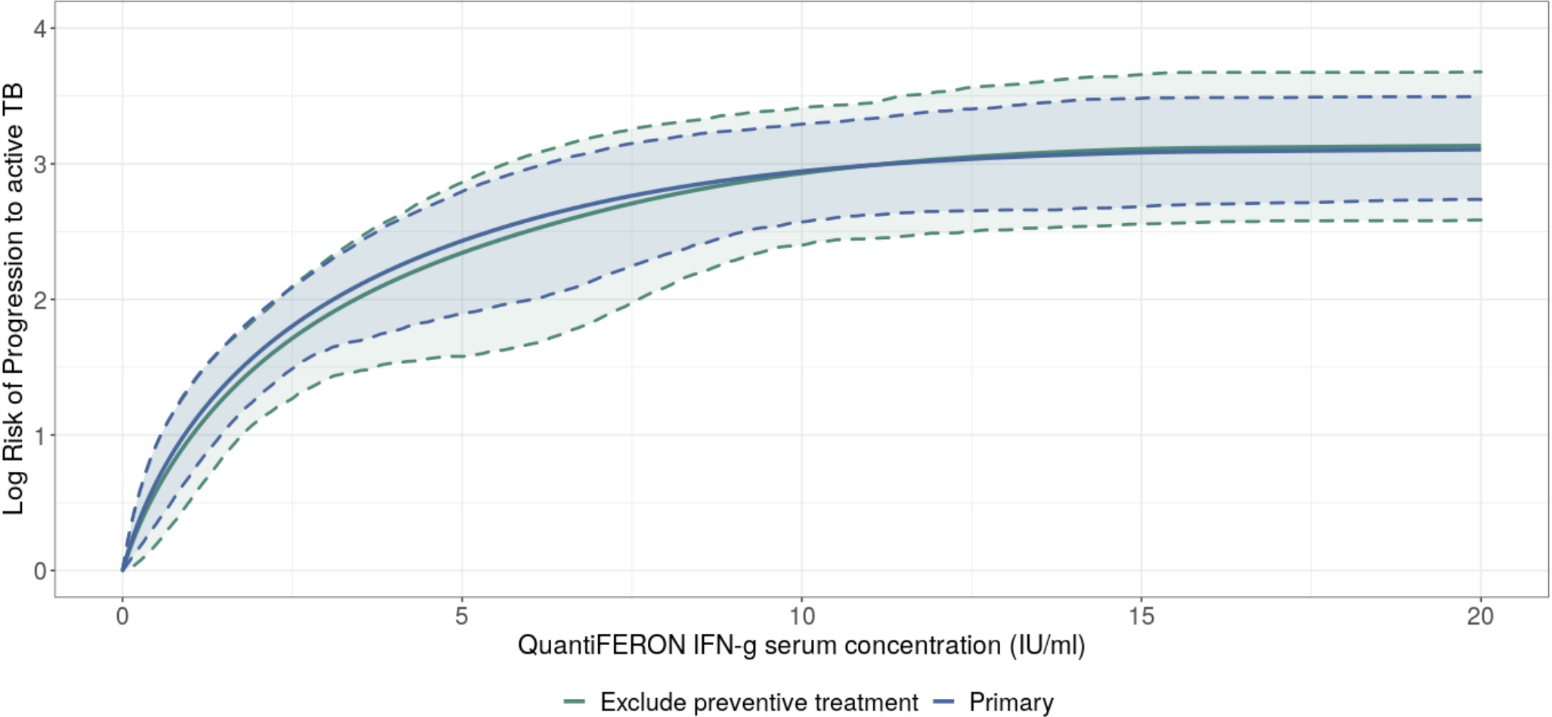


**Estimating the relative risks of developing active TB by interferon gamma levels**

Our mixed-effects regression model can be represented as the following:

$$ln(relative risk)=(\frac{\ln\left( 1+X_{comp}\beta\right)-\ln\left( 1+X_{ref}\beta\right)}{X_{comp}-X_{ref}}+u)(X_{comp}-X_{ref})$$

Where $X_{comp}$ and $X_{ref}$ are the design matrices for the comparison and reference groups; $\beta$ is a vector of regression coefficients; $u$ is the study random effect assuming a random slope.

The risk function occurring within the design matrices is defined by:

$$relative risk\left( s,e,\beta\right)=\sum_{i=1}^{m} \beta_{i}\frac{1}{e-s}\int_{s}^{e} f_{i} \left( t \right)dt$$

where $s$ is the lower bound of interferon gamma levels (IU/ml) for a data point; $e$ is the upper bound of interferon gamma levels (IU/ml) for a data point; $\beta$ is the regression coefficient at $i$. Particularly, we use a set of spline bases to parametrize the relative risk function of interferon gamma levels. Each $f_{i}$ is one of the bases and $\beta_{i}$ are the corresponding coefficients.

**PRISMA-P 2015 Checklist**

| **Section/topic** | **#** | **Checklist item** | **Information reported** | | **Criteria location** |
| --- | --- | --- | --- | --- | --- |
|  |  |  | **Yes** | **No** |  |
| **ADMINISTRATIVE INFORMATION** | | | | | |
| **Title** | | | | | |
| Identification | 1a | Identify the report as a protocol of a systematic review |  |  | Title page |
| Update | 1b | If the protocol is for an update of a previous systematic review, identify as such |  |  | N/A |
| **Registration** | 2 | If registered, provide the name of the registry (e.g., PROSPERO) and registration number in the Abstract |  |  | N/A |
| **Authors** | | | | | |
| Contact | 3a | Provide name, institutional affiliation, and e-mail address of all protocol authors; provide physical mailing address of corresponding author |  |  | Title page |
| Contributions | 3b | Describe contributions of protocol authors and identify the guarantor of the review |  |  | Author Contributions section |
| **Amendments** | 4 | If the protocol represents an amendment of a previously completed or published protocol, identify as such and list changes; otherwise, state plan for documenting important protocol amendments |  |  | N/A |
| **Support** | | | | | |
| Sources | 5a | Indicate sources of financial or other support for the review |  |  | Funding section |
| Sponsor | 5b | Provide name for the review funder and/or sponsor |  |  | Funding section |
| Role of sponsor/funder | 5c | Describe roles of funder(s), sponsor(s), and/or institution(s), if any, in developing the protocol |  |  | Funding section |
| **INTRODUCTION** | | | | | |
| **Rationale** | 6 | Describe the rationale for the review in the context of what is already known |  |  | Introduction section:  Paragraph 2 & 3 |
| **Objectives** | 7 | Provide an explicit statement of the question(s) the review will address with reference to participants, interventions, comparators, and outcomes (PICO) |  |  | Introduction section:  Paragraph 3 |
| **METHODS** | | | | | |
| **Eligibility criteria** | 8 | Specify the study characteristics (e.g., PICO, study design, setting, time frame) and report characteristics (e.g., years considered, language, publication status) to be used as criteria for eligibility for the review |  |  | Methods section:  Paragraph 2 |
| **Information sources** | 9 | Describe all intended information sources (e.g., electronic databases, contact with study authors, trial registers, or other grey literature sources) with planned dates of coverage |  |  | Methods section:  Paragraph 1 |
| **Search strategy** | 10 | Present draft of search strategy to be used for at least one electronic database, including planned limits, such that it could be repeated |  |  | Supplements  Table S2 |
| ***STUDY RECORDS*** | | | | | |
| Data management | 11a | Describe the mechanism(s) that will be used to manage records and data throughout the review |  |  | N/A |
| Selection process | 11b | State the process that will be used for selecting studies (e.g., two independent reviewers) through each phase of the review (i.e., screening, eligibility, and inclusion in meta-analysis) |  |  | Methods section:  Paragraph 2 |
| Data collection process | 11c | Describe planned method of extracting data from reports (e.g., piloting forms, done independently, in duplicate), any processes for obtaining and confirming data from investigators |  |  | Methods section:  Paragraph 3 |
| **Data items** | 12 | List and define all variables for which data will be sought (e.g., PICO items, funding sources), any pre-planned data assumptions and simplifications |  |  | Methods section:  Paragraph 3 |
| **Outcomes and prioritization** | 13 | List and define all outcomes for which data will be sought, including prioritization of main and additional outcomes, with rationale |  |  | Methods section:  Paragraph 3 |
| **Risk of bias in individual studies** | 14 | Describe anticipated methods for assessing risk of bias of individual studies, including whether this will be done at the outcome or study level, or both; state how this information will be used in data synthesis |  |  | Methods section:  Paragraph 4 |
| ***DATA*** | | | | | |
| **Synthesis** | 15a | Describe criteria under which study data will be quantitatively synthesized |  |  | Methods section:  Paragraph 5 |
|  | 15b | If data are appropriate for quantitative synthesis, describe planned summary measures, methods of handling data, and methods of combining data from studies, including any planned exploration of consistency (e.g., *I* ^2^, Kendall’s tau) |  |  | Methods section:  Paragraph 5 |
|  | 15c | Describe any proposed additional analyses (e.g., sensitivity or subgroup analyses, meta-regression) |  |  | Methods section:  Paragraph 6 |
|  | 15d | If quantitative synthesis is not appropriate, describe the type of summary planned |  |  | N/A |
| **Meta-bias(es)** | 16 | Specify any planned assessment of meta-bias(es) (e.g., publication bias across studies, selective reporting within studies) |  |  | Methods section:  Paragraph 6 |
| **Confidence in cumulative evidence** | 17 | Describe how the strength of the body of evidence will be assessed (e.g., GRADE) |  |  | Discussion section:  Paragraph 1 |

**References**

1. Abdulkareem FN, Merza MA, Salih AM. First insight into latent tuberculosis infection among household contacts of tuberculosis patients in Duhok, Iraqi Kurdistan: using tuberculin skin test and QuantiFERON-TB Gold Plus test. Int J Infect Dis [Internet]. 2020 Jul;96:97–104. Available from: https://linkinghub.elsevier.com/retrieve/pii/S1201971220302009

2. Ahmed A, Feng P-JI, Gaensbauer JT, Reves RR, Khurana R, Salcedo K, et al. Interferon-γ Release Assays in Children <15 Years of Age. Pediatrics [Internet]. 2020 Jan;145(1):e20191930. Available from: http://pediatrics.aappublications.org/lookup/doi/10.1542/peds.2019-1930

3. Aichelburg MC, Rieger A, Breitenecker F, Pfistershammer K, Tittes J, Eltz S, et al. Detection and Prediction of Active Tuberculosis Disease by a Whole‐Blood Interferon‐γ Release Assay in HIV‐1–Infected Individuals. Clin Infect Dis [Internet]. 2009 Apr;48(7):954–62. Available from: https://academic.oup.com/cid/article-lookup/doi/10.1086/597351

4. Altet N, Dominguez J, Souza-Galvão M-L de, Jiménez-Fuentes MÁ, Milà C, Solsona J, et al. Predicting the Development of Tuberculosis with the Tuberculin Skin Test and QuantiFERON Testing. Ann Am Thorac Soc [Internet]. 2015 May;12(5):680–8. Available from: http://www.atsjournals.org/doi/10.1513/AnnalsATS.201408-394OC

5. Andrews JR, Nemes E, Tameris M, Landry BS, Mahomed H, McClain JB, et al. Serial QuantiFERON testing and tuberculosis disease risk among young children: an observational cohort study. Lancet Respir Med [Internet]. 2017 Apr;5(4):282–90. Available from: https://linkinghub.elsevier.com/retrieve/pii/S2213260017300607

6. Bergot E, Haustraete E, Malbruny B, Magnier R, Salaün M-A, Zalcman G. Observational Study of QuantiFERON®-TB Gold In-Tube Assay in Tuberculosis Contacts in a Low Incidence Area. Herrmann JL, editor. PLoS One [Internet]. 2012 Aug 24;7(8):e43520. Available from: https://dx.plos.org/10.1371/journal.pone.0043520

7. Torres Costa J, Silva R, Ringshausen FC, Nienhaus A. Screening for tuberculosis and prediction of disease in Portuguese healthcare workers. J Occup Med Toxicol [Internet]. 2011;6(1):19. Available from: http://occup-med.biomedcentral.com/articles/10.1186/1745-6673-6-19

8. Diel R, Loddenkemper R, Niemann S, Meywald-Walter K, Nienhaus A. Negative and Positive Predictive Value of a Whole-Blood Interferon-γ Release Assay for Developing Active Tuberculosis. Am J Respir Crit Care Med [Internet]. 2011 Jan;183(1):88–95. Available from: http://www.atsjournals.org/doi/abs/10.1164/rccm.201006-0974OC

9. Doyle JS, Bissessor M, Denholm JT, Ryan N, Fairley CK, Leslie DE. Latent Tuberculosis Screening Using Interferon-Gamma Release Assays in an Australian HIV-Infected Cohort. JAIDS J Acquir Immune Defic Syndr [Internet]. 2014 May;66(1):48–54. Available from: http://content.wkhealth.com/linkback/openurl?sid=WKPTLP:landingpage&an=00126334-201405010-00007

10. Giri P, Basu S, Sargeant T, Rimmer A, Pirzada O, Adisesh A. Pre-placement screening for tuberculosis in healthcare workers. Occup Med (Chic Ill) [Internet]. 2014 Oct 1;64(7):524–9. Available from: https://academic.oup.com/occmed/article-lookup/doi/10.1093/occmed/kqu107

11. Gupta RK, Lipman M, Jackson C, Sitch AJ, Southern J, Drobniewski F, et al. Quantitative IFN-γ Release Assay and Tuberculin Skin Test Results to Predict Incident Tuberculosis. A Prospective Cohort Study. Am J Respir Crit Care Med [Internet]. 2020 Apr 15;201(8):984–91. Available from: https://www.atsjournals.org/doi/10.1164/rccm.201905-0969OC

12. Haldar P, Thuraisingam H, Patel H, Pereira N, Free RC, Entwisle J, et al. Single-step QuantiFERON screening of adult contacts: a prospective cohort study of tuberculosis risk. Thorax [Internet]. 2013 Mar;68(3):240–6. Available from: http://thorax.bmj.com/lookup/doi/10.1136/thoraxjnl-2011-200956

13. Harstad I, Winje BA, Heldal E, Oftung F, Jacobsen GW. Predictive values of QuantiFERON-TB Gold testing in screening for tuberculosis disease in asylum seekers. Int J Tuberc lung Dis [Internet]. 2010 Sep;14(9):1209–11. Available from: http://www.ncbi.nlm.nih.gov/pubmed/20819271

14. Jonsson J, Westman A, Bruchfeld J, Sturegård E, Gaines H, Schön T. A borderline range for Quantiferon Gold In-Tube results. Shams (Amir) Homayoun, editor. PLoS One [Internet]. 2017 Nov 2;12(11):e0187313. Available from: https://dx.plos.org/10.1371/journal.pone.0187313

15. Joshi R, Narang U, Zwerling A, Jain D, Jain V, Kalantri S, et al. Predictive value of latent tuberculosis tests in Indian healthcare workers: a cohort study. Eur Respir J [Internet]. 2011 Dec 1;38(6):1475–7. Available from: http://erj.ersjournals.com/cgi/doi/10.1183/09031936.00014611

16. Kruczak K, Duplaga M, Sanak M, Cmiel A, Mastalerz L, Sladek K, et al. Comparison of IGRA tests and TST in the diagnosis of latent tuberculosis infection and predicting tuberculosis in risk groups in Krakow, Poland. Scand J Infect Dis [Internet]. 2014 Sep 30;46(9):649–55. Available from: http://www.tandfonline.com/doi/full/10.3109/00365548.2014.927955

17. Lee S, Lee JE, Kang JS, Lee SO, Lee SH. Long-term performance of the IGRA to predict and prevent active tuberculosis development in HIV-infected patients. Int J Tuberc Lung Dis [Internet]. 2019 Apr 1;23(4):422–7. Available from: https://www.ingentaconnect.com/content/10.5588/ijtld.18.0198

18. Lu P, Liu Q, Zhou Y, Martinez L, Kong W, Ding X, et al. Predictors of Discordant Tuberculin Skin test and QuantiFERON-TB Gold In-Tube Results in Eastern China: A Population-based, Cohort Study. Clin Infect Dis [Internet]. 2020 May 5;15(8):1056–61. Available from: http://www.ncbi.nlm.nih.gov/pubmed/21740668

19. Mahomed H, Hawkridge T, Verver S, Abrahams D, Geiter L, Hatherill M, et al. The Tuberculin Skin Test versus QuantiFERON TB Gold in Predicting Tuberculosis Disease in an Adolescent Cohort Study in South Africa. Pai M, editor. PLoS One [Internet]. 2011 Mar 29;6(3):e17984. Available from: https://dx.plos.org/10.1371/journal.pone.0017984

20. Nienhaus A, Costa JT. Screening for tuberculosis and the use of a borderline zone for the interpretation of the interferon-γ release assay (IGRA) in Portuguese healthcare workers. J Occup Med Toxicol [Internet]. 2013;8(1):1. Available from: http://occup-med.biomedcentral.com/articles/10.1186/1745-6673-8-1

21. Noorbakhsh S, Mousavi J, Barati M, Shamshiri AR, Shekarabi M, Tabatabaei A, et al. Evaluation of an interferon-gamma release assay in young contacts of active tuberculosis cases. East Mediterr Heal J [Internet]. 2011 Sep;17(9):714–8. Available from: http://www.ncbi.nlm.nih.gov/pubmed/22259925

22. Ringshausen FC, Nienhaus A, Schablon A, Schlösser S, Schultze-Werninghaus G, Rohde G. Predictors of persistently positive Mycobacterium-tuberculosis-specific interferon-gamma responses in the serial testing of health care workers. BMC Infect Dis [Internet]. 2010 Jul 23;10:220. Available from: http://www.ncbi.nlm.nih.gov/pubmed/20653946

23. Rose W, Kitai I, Kakkar F, Read SE, Behr MA, Bitnun A. Quantiferon Gold-in-tube assay for TB screening in HIV infected children: influence of quantitative values. BMC Infect Dis [Internet]. 2014 Sep 23;14(1):516. Available from: https://bmcinfectdis.biomedcentral.com/articles/10.1186/1471-2334-14-516

24. Santin M, Casas S, Saumoy M, Andreu A, Moure R, Alcaide F, et al. Detection of latent tuberculosis by the tuberculin skin test and a whole-blood interferon-γ release assay, and the development of active tuberculosis in HIV-seropositive persons. Diagn Microbiol Infect Dis [Internet]. 2011 Jan;69(1):59–65. Available from: https://linkinghub.elsevier.com/retrieve/pii/S0732889310003652

25. Schablon A, Peters C, Diel R, Diner G, Anske U, Pankow W, et al. Serial IGRA testing of trainees in the healthcare sector in a country with low incidence for tuberculosis - a prospective cohort study. GMS Hyg Infect Control [Internet]. 2013;8(2):Doc17. Available from: http://www.ncbi.nlm.nih.gov/pubmed/24327943

26. Sharma SK, Vashishtha R, Chauhan LS, Sreenivas V, Seth D. Comparison of TST and IGRA in Diagnosis of Latent Tuberculosis Infection in a High TB-Burden Setting. Hasnain SE, editor. PLoS One [Internet]. 2017 Jan 6;12(1):e0169539. Available from: https://dx.plos.org/10.1371/journal.pone.0169539

27. Tsou P, Huang W-C, Huang C, Lin C, Wu K, Hsu J, et al. Quantiferon TB-Gold conversion can predict active tuberculosis development in elderly nursing home residents. Geriatr Gerontol Int [Internet]. 2015 Oct;15(10):1179–84. Available from: http://doi.wiley.com/10.1111/ggi.12416

28. Verhagen LM, Maes M, Villalba JA, D’Alessandro A, Rodriguez LP, España MF, et al. Agreement between QuantiFERON-TB Gold In-Tube and the tuberculin skin test and predictors of positive test results in Warao Amerindian pediatric tuberculosis contacts. BMC Infect Dis [Internet]. 2014 Dec 11;14(1):383. Available from: https://bmcinfectdis.biomedcentral.com/articles/10.1186/1471-2334-14-383

29. A. Whitaker J, Mirtskhulava V, Kipiani M, Harris DA, Tabagari N, Kempker RR, et al. Prevalence and Incidence of Latent Tuberculosis Infection in Georgian Healthcare Workers. Ruhwald M, editor. PLoS One [Internet]. 2013 Mar 25;8(3):e58202. Available from: https://dx.plos.org/10.1371/journal.pone.0058202

30. Winje BA, White R, Syre H, Skutlaberg DH, Oftung F, Mengshoel AT, et al. Stratification by interferon-γ release assay level predicts risk of incident TB. Thorax [Internet]. 2018 Jul;73(7):652–61. Available from: http://thorax.bmj.com/lookup/doi/10.1136/thoraxjnl-2017-211147

31. Yoshiyama T, Harada N, Higuchi K, Saitou M, Kato S. Use of the QuantiFERON ® -TB Gold in Tube test for screening TB contacts and predictive value for active TB. Infect Dis (Auckl) [Internet]. 2015 Aug 3;47(8):542–9. Available from: http://www.tandfonline.com/doi/full/10.3109/23744235.2015.1026935

32. Yoshiyama T, Harada N, Higuchi K, Sekiya Y, Uchimura K. Use of the QuantiFERON-TB Gold test for screening tuberculosis contacts and predicting active disease. Int J Tuberc lung Dis [Internet]. 2010 Jul;14(7):819–27. Available from: http://www.ncbi.nlm.nih.gov/pubmed/20550763

33. Zellweger J-P, Sotgiu G, Block M, Dore S, Altet N, Blunschi R, et al. Risk Assessment of Tuberculosis in Contacts by IFN-γ Release Assays. A Tuberculosis Network European Trials Group Study. Am J Respir Crit Care Med [Internet]. 2015 May 15;191(10):1176–84. Available from: http://www.atsjournals.org/doi/10.1164/rccm.201502-0232OC

34. Zenner D, Loutet MG, Harris R, Wilson S, Ormerod LP. Evaluating 17 years of latent tuberculosis infection screening in north-west England: a retrospective cohort study of reactivation. Eur Respir J [Internet]. 2017 Jul 27;50(1):1602505. Available from: http://erj.ersjournals.com/lookup/doi/10.1183/13993003.02505-2016

35. Kashangura R, Jullien S, Garner P, Johnson S. MVA85A vaccine to enhance BCG for preventing tuberculosis. Cochrane database Syst Rev [Internet]. 2019;4:CD012915. Available from: http://www.ncbi.nlm.nih.gov/pubmed/31038197
